# Engineering Bacterial Cellulose by Synthetic Biology

**DOI:** 10.3390/ijms21239185

**Published:** 2020-12-02

**Authors:** Amritpal Singh, Kenneth T. Walker, Rodrigo Ledesma-Amaro, Tom Ellis

**Affiliations:** 1 Imperial College Centre for Synthetic Biology, Imperial College London, London SW7 2AZ, UK; a.singh16@imperial.ac.uk (A.S.); k.walker16@imperial.ac.uk (K.T.W.); r.ledesma-amaro@imperial.ac.uk (R.L.-A.); 2 Department of Bioengineering, Imperial College London, London SW7 2AZ, UK

**Keywords:** bacterial cellulose, synthetic biology, metabolic engineering, biomaterials

## Abstract

Synthetic biology is an advanced form of genetic manipulation that applies the principles of modularity and engineering design to reprogram cells by changing their DNA. Over the last decade, synthetic biology has begun to be applied to bacteria that naturally produce biomaterials, in order to boost material production, change material properties and to add new functionalities to the resulting material. Recent work has used synthetic biology to engineer several *Komagataeibacter* strains; bacteria that naturally secrete large amounts of the versatile and promising material bacterial cellulose (BC). In this review, we summarize how genetic engineering, metabolic engineering and now synthetic biology have been used in *Komagataeibacter* strains to alter BC, improve its production and begin to add new functionalities into this easy-to-grow material. As well as describing the milestone advances, we also look forward to what will come next from engineering bacterial cellulose by synthetic biology.

## 1. Introduction

If you look up from this text and look around, it is very likely that you will see cellulose and cellulose-based products within reach. Thanks to its ubiquity in the plant kingdom and its use in so many everyday materials and textiles, it is hard not to see cellulose everywhere. Indeed if you zoom down on the smallest life forms on the planet, bacteria, you can often see cellulose too. It is made and used by many microbes as part of their protective biofilms or is a component of their cell wall [1].

The most prodigious bacterial producers of cellulose are found within the *Acetobacteraceae* family, often known as Acetic Acid Bacteria (AAB). These rod-shaped bacteria are gram-negative obligate aerobes and produce copious amounts of acetic acid by metabolizing carbon sources into acetic acid and ethanol via their oxidative fermentation pathways [2]. They are widespread and known for their industrial applications in food and beverage production particularly in the production of vinegar and kombucha tea [3].

The species of two genera of this family—*Gluconacetobacter* and *Komagataeibacter*—are not only highly acid-resistant and efficient in oxidizing ethanol into acetic acid but they also secrete cellulose in impressive quantities [4]. Because of this, at least five *Komagataeibacter* species have become the focus of significant research and industry: *K. xylinus, K. hansenii, K. rhaeticus, K. europaeus* and *K. medellinensis* [5]. Strains of these species are the most efficient producers of cellulose of all bacteria and the model organisms for bacterial cellulose research and applications [6].

Bacterial cellulose (BC) is a polymer of β-(1→4)-D-glucose monomers, which forms when glucose is modified to attach a uridine diphosphate (UDP) group and the subsequent UDP-glucose is then polymerized by the cellulose synthase complex in the cell’s inner membrane. As these polymers form, they are immediately secreted out from the cell and self-assemble into fibrils and then into larger cellulose fibers [7]. The fibers formed from a growing population of these cells intertwine to form a BC lattice and as this lattice increases in volume and density (usually at the air-liquid interface of a growing culture) it forms a visible material mat known as a ‘pellicle.’

The bacterial cellulose produced by *Komagataeibacter* species has highly desirable material properties for advanced and specialty applications. Unlike the cellulose found in plants, it is formed of long continuous polymers with very little branching and it is devoid of heterologous polymers such as hemicellulose, pectin and lignin [8]. Due to this homogeneity, the cellulose polymers within BC can tightly pack together giving unique properties such as high crystallinity and high tensile strength [9,10,11]. The lack of lignin and other contaminants also provides biocompatibility and better biodegradability. Indeed, studies assessing the tissue reaction of subcutaneous implants of BC in rodents found no evidence of foreign body rejection [12]. The arrangement and abundance of hydroxyl groups on the cellulose chains enable an unusually high number of water molecules to bind within the BC fibers. As such hydrated BC has hydrogel-like properties due to its very high (98% *w*/*v*) water content [9]. Thanks to all these properties and more, BC made by *Komagataeibacter* has been put to use in a wide range of applications from those in healthcare and biomedicine to those in consumer electronics [13,14,15]. As interest and demand for this material rises, researchers and industry are increasingly looking to improve the biotechnology of BC production from bacteria.

Microbial biotechnology has been around for decades or even centuries depending on how you define it. However, in the last 20 years it has been transformed thanks to a slew of new tools and technologies for genome sequencing and genetic engineering. For the major industrial microbes—yeasts and *Escherichia coli*—the most transformative changes during this time have been due to the new field of synthetic biology. Synthetic biology takes an engineering approach to genetically modifying a cell within a sequenced genome, treating its DNA as reprogrammable code that can be synthesized, edited and inserted into cells to improve specific cell functions or add new functionalities [16]. By taking cues from other engineering disciplines and making use of modularity, standardization and mathematical design approaches, synthetic biology has greatly accelerated what is possible for metabolic and genetic engineering in key microbes [17].

In the last decade, what is possible in synthetic biology for *E. coli* and for yeasts has started to become possible in other microbes. Indeed a few pioneering groups have now begun to apply synthetic biology methods to *Komagataeibacter* in the hope of engineering strains of bacteria that produce higher yields of BC, make BC with improved quality or produce BC with modified properties or new functionalities that make it of high value for new research and application areas. In this review we will summarize how past work in genetic engineering of BC-producing bacteria has now led to the first successes in using synthetic biology in *Komagataeibacter* and in producing BC-based materials.

## 2. Genetic Engineering of *Acetobacteraceae*

Genetic engineering has been one of the main tools used in industrial biotechnology and research since the 1980s but until recently has not been widely-used to aid or alter the production of bacterial cellulose from *Acetobacteraceae.* In part this is due to the relative obscurity of these bacteria in biotechnology research but it is also because BC researchers and industrial producers have demonstrated many non-genetic interventions that can very effectively improve BC yields, change the polymer qualities and impart new functionalities into the grown material. These methods have been reviewed extensively elsewhere and generally require changes to the growth media and culturing conditions or require the addition of chemical and polymer additives as the material is growing [18].

The most basic genetic interventions used to improve or alter BC production simply focus on strain selection. The different *Komagataeibacter* species used in industrial BC production have varied yields in different growth media and conditions and each make BC with different material properties like cellulose purity and crystallinity [4,10]. Selection coupled with natural mutation in the culturing environment or in the lab has led to key strains being identified for each species that perform best at producing high quality, high yield BC [5]. This untargeted approach of strain mutation and selection, while successful, does not have the speed and precision of genetic engineering. Furthermore it is entirely unsuitable when researchers want to bring new functionalities into *Acetobacteraceae* that need to be encoded by genes unlikely to be found naturally in these bacteria.

The first major example of genetic engineering in BC-producing bacteria was the expression of a high affinity mutant of mung bean sucrose synthase in a *K. xylinus* strain in 1999. The simple addition of DNA encoding expression of this enzyme led to enhanced BC production when sucrose was included in the growth media, providing a route to higher yields of BC materials with lower feedstock costs [19]. The coding sequence (CDS) of the mutant gene was simply cloned into a plasmid downstream of a lac promoter and this was selected for in the bacteria using ampicillin.

Two further examples of genetic engineering in the following decade showed alternative ways to improve BC yields from cheap feedstocks. The *lacZ* gene from *E. coli* was inserted into the *K. xylinus* genome to enable lactose to be utilized when BC is grown from whey [20] and a hemoglobin gene from *Vitreoscilla* was expressed from the *E. coli bla* promoter on a broad-host range plasmid in *K. xylinus* to aid oxygenation of the cells and thus boost BC yields in shaken cultures [21].

**Figure 1:**
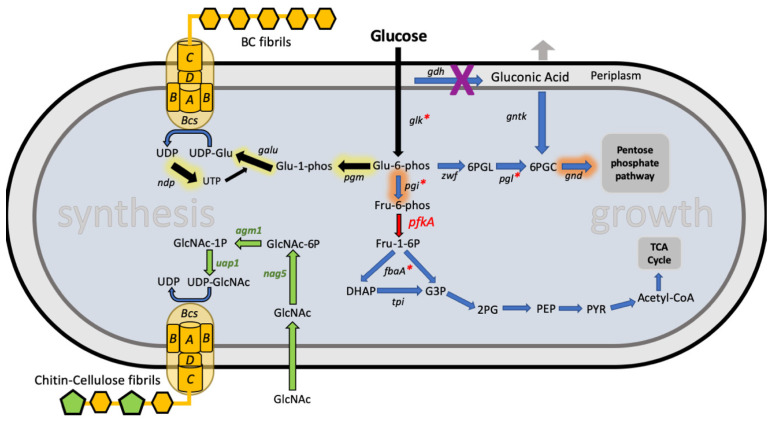
The metabolic pathway to bacterial cellulose biosynthesis in *Komagataeibacter* and example metabolic engineering interventions made in key papers. Native pathways from glucose to central carbon metabolism (*growth*) are shown as blue arrows. The native pathway to cellulose biosynthesis via the BcsABCD machinery (*synthesis*) is shown as black arrows. Heterologous expression of a 3-gene GlcNAc utilization pathway by Yadav et al. to produce chitin-cellulose co-polymers is shown as green arrows [22]. Interventions made by Gwon et al. to boost cellulose production are indicated in red; red arrow shows insertion of a *pfkA* enzyme, red asterisks show altered regulation of these genes via overexpression of the cAMP receptor protein (CRP) regulator [24]. Interventions made by Hur et al. to boost cellulose production are highlighted in yellow: expression of enzymes encoded by the *galU, ndp* and *pgm* genes are optimized by RBS tuning [25]. Interventions made by Jang et al. are highlighted in orange: heterologous expression is used to boost enzyme levels encoded by *pgi* and *gnd* [26]. Genomic deletion of the *gdh* gene by Liu et al. to reduce gluconic acid bi-product formation is shown as a purple X [27]. Metabolite abbreviations; Glu-6-phos: glucose-6-phosphate; 6PGL: 6-phosphogluconolactone; 6PGC: 6-phosphogluconate; Fru-6-phos: fructose-6-phosphate; Fru-1-6P: fructose-1,6-diphosphate; DHAP: dihydroxyacetone phosphate; G3P: glyceraldehyde-3-phosphate; 2PG: 2-phosphoglyceric acid; PEP: phosphoenol pyruvate; PYR: pyruvate; Glu-1-phos: glucose-1-phosphate; UTP: uridine triphosphate; UDP: uridine diphosphate. UDP-Glu: UDP-glucose; GlcNac: *N*-acetylglucosamine; GlcNAc-1P: *N*-acetylglucosamine-1-phosphate; GlcNAc-6P: *N*-acetylglucosamine-6-phosphate; UDP-GlcNAc: UDP-*N*-acetylglucosamine.

While these and other genetic engineering studies have focused only on the addition or deletion of one gene in order to improve BC production, a study published in 2010 went much further. Using the same plasmid vector and promoter as used in the hemoglobin study, Yadav et al. introduced an operon of 3 genes from the yeast *Candida albic*ans into *K. xylinus* [22]. Together these genes encode a metabolic pathway to enable the monomer of chitin, *N-*acetylglucosamine (GlcNAc), to be converted into UDP-GlcNAc inside the bacteria. The UDP-GlcNAc monomers then intermix with the UDP-glucose molecules and both end up being polymerized together by the cell’s cellulose biosynthesis machinery (Figure 1). The result is an engineered strain that when fed GlcNAc as well as glucose, produces a novel cellulose-chitin copolymer. As chitin is susceptible to degradation by animal lysozymes, the co-polymer produced by this method can be naturally degraded when implanted in vivo, unlike cellulose which cannot. This work makes possible the creation of in vivo BC structures, such as stents and venal prosthetics, which can be naturally degraded by the body and do not require surgical removal [22,23].

The Yadav et al. study demonstrated that genetic engineering in *K. xylinus* could go beyond just the expression of single genes and that intracellular metabolism could be augmented by the addition of extra enzymes [22]. Effectively this demonstrated that metabolic engineering was possible in *Acetobacteraceae.*

## 3. Metabolic Engineering for Enhancing BC Production

Metabolic engineering is a specific use of genetic engineering in industrial biotechnology where enzymes (and occasionally gene regulatory proteins) are expressed in cells in an effort to redirect the metabolism of an organism to improve yields and quality of a biochemical product [28]. In recent years, metabolic engineering has become an advanced discipline, often using mathematical models of cellular metabolism to predict worthwhile genetic interventions ahead of lab work and then making use of synthetic biology tools to do precision genetic engineering to achieve its goals [29,30]. A key tool for designing and evaluating metabolic engineering is flux-balance analysis (FBA) [31]. This is a mathematical approach for analyzing the flow of metabolites through a metabolic network and is used computationally to identify ways to enhance yields of target metabolites and predict the effects of introducing new enzymes or changing the levels of native enzymes in the cell [32].

FBA has been used to evaluate and understand the metabolism of both acetic acid producing and BC-producing strains of *Acetobacteraceae* bacteria [33,34]. This has largely been done to direct industrial users of these strains to rationally select new culture media and growth conditions that can better direct the flux of metabolism to greater yields of the desired product. Indeed, it has not been until recently that FBA and genetic engineering have finally been combined to metabolically engineer an acetobacter. This was first achieved in 2017, when an industrial acetic acid producer strain of *Acetobacter pasteurianus* was genetically modified to improve acetic acid yields [35]. FBA combined with plasmid-based overexpression of subunits adhA and adhB of pyrroquinoline quinone-dependent alcohol dehydrogenase (PQQ-ADH), led the researchers to understand how to improve the ethanol oxidase respiratory chain in their cells, leading to more ethanol being converted into the acetic acid product [35].

While this work demonstrated FBA-guided metabolic engineering in an acetobacter, it was not until 2019 that metabolic engineering was shown to be a route for improving production yields of BC (Figure 1). In an impressive body of work by a team of researchers based at Samsung and at KAIST in South Korea, a genome-scale metabolic model of a *K. xylinus* strain was first constructed by taking data from genome sequencing and metabolome analysis of this strain [26]. The team based at KAIST first used this model to explore increasing BC yields by overexpressing *pgi* and *gnd* genes from *E. coli* or *Corynebacterium glutamicum* in their strain and achieved a doubling (from 1.46 g/L to 3.15 g/L) of BC production through this approach [26]. Then in collaboration with Samsung they explored an alternative route that pushed strain BC production even higher up to 4.5 g/L (corresponding to a 28.4% yield from glucose) [24]. The key to this optimization was the observation from their *‘Koma’* metabolic model that intracellular levels of adenosine triphosphate (ATP) are crucial for determining cellulose yield. A major enzyme in the native pathways of *K. xylinus*, glucose-6-phosphate (g6p) dehydrogenase (encoded by the *zwf* gene), is a key branch point for determining whether glucose is metabolized or used for cellulose biosynthesis. The enzyme is strongly inhibited by high ATP levels and so increasing intracellular ATP will push more glucose into cellulose production.

To achieve increased ATP levels, they added a plasmid encoding heterologous expression of phosphofructokinase from the *E. coli pfkA* gene. *Komagataeibacter* lack this critical enzyme for glycolysis and instead metabolize most glucose via the pentose phosphate pathway. Introducing this gene established the glycolysis pathway and boosted ATP levels by 4-fold. As the model predicted, this led to a boost in growth and in cellulose production levels too [24]. The authors then tried to improve on this further, first by introducing a plasmid encoding other glycolysis genes constitutively expressed from the strong tac promoter in order to try to boost this pathway further. However, this was not successful in improving production. Instead, a change in regulation of metabolism was made. The *E. coli* cAMP receptor protein (CRP) was introduced by adding its gene into the *pfkA-*expressing plasmid. CRP is a master regulator transcription factor and is known to positively regulate the expression of glucose metabolizing genes in *E. coli*, including those of glycolysis. Its addition into *K. xylinus*, alongside *pfkA* further boosted cellulose production but perhaps more importantly nearly halved the amount of glucose that was lost to the unwanted by-product, gluconic acid. This model-guided metabolic engineering thus achieved a high increase in the yield of cellulose from glucose, increasing it 3-fold from 9.5% to 28% [24].

Interestingly, achieving such high yields of BC via this approach came at a cost-non-producing (*Cel^-^*) mutant strains would regularly arise during the production phase (stirred batch fermentation) and take over the culture, consuming the sugar feedstock and dividing while not making any BC. The team used genome sequencing to investigate and identified that the *Cel^-^* mutation was regularly caused by an insertion sequence (IS) transposable element jumping into the bcsA gene that encodes the cellulose synthase machinery [25]. They then used genetic engineering to modify the DNA sequence of this key gene to reduce the ability of common IS elements to insert into it and disrupt its function. This engineering led to a 1.7-fold increase in productivity compared to a control strain [25].

## 4. Synthetic Biology Toolkits for Bringing New Functionality to Bacterial Cellulose

Synthetic biology goes beyond the genetic engineering approaches described in the work above by treating DNA not just as genetic information that can be mutated or added into cells but seeing it fundamentally as a modular code that can be treated like a programming language that can open up new possibilities in biotechnology [36]. Since its emergence in 2000, it has had a major impact on the engineering and industrial application of many organisms, most notably the model microbes *E. coli* and *Saccharomyces cerevisiae* (Baker’s yeast). The rich information available at the genome, transcriptome, proteome and metabolome levels for these organisms have made them the go-to cells for creating modular DNA part libraries and rapid cloning toolkits that can quickly and easily combine these DNA parts into novel genetic programs [37,38]. Synthetic biology projects in these microbes regularly now involve the design and construction of synthetic DNA programs 10 s and 100 s of kilobases in length, often containing dozens of modular DNA parts and typically with many design variants built and tested in parallel [39,40].

Genome, transcriptome and metabolome data have only been generated quite recently for BC-producing organisms like *K. xylinus*. As such it is unsurprising that synthetic biology has been slower to take off for these bacteria. Nonetheless significant progress has been made in the last 5 years to introduce and use modular DNA programming for engineering BC-producing bacteria. This has resulted in a handful of publications that now use synthetic biology approaches to engineer cells to have new functionalities and to produce BC with modified material properties.

Florea et al. (2016) were the first to bring the key principles of modular DNA engineering to BC-producing bacteria, when they described a ‘toolkit’ of standardized, modular DNA parts that could be used for genetic programming of controlled gene expression in the bacteria as they grow and produce cellulose pellicles [41]. Like many other innovations in synthetic biology, their work began as a student project for the international Genetically Engineered Machines (iGEM) competition. They isolated, characterized and sequenced their own strain of *Komagataeibacter* taken from a kombucha tea pellicle. This was revealed to be a *K. rhaeticus* species bacteria that proved more amenable to plasmid-based genetic modification than more established *K. xylinus* and *K. hansenii* strains [42].

Using this transformable bacteria, they were able to clone and characterize the performance of dozens of modular DNA parts including fluorescent protein reporter genes, terminator sequences, synthetic promoters of different strength and regulated promoters controlled by transcription factors that are chemically-inducible by external application of the antibiotic analogue anhydrotetracycline (aTC) or the quorum sensing molecule acyl-homoserine lactone (AHL) [41]. These modular DNA parts were designed to a standard cloning format called BioBricks [43], which meant that they could be quickly assembled together in many different combinations in plasmids built in *E. coli* that could then be shuttled into the *K. rhaeticus* strain. Crucially the authors described and tested many different plasmid systems to find those that worked best in their bacteria, settling on two modular synthetic vectors from the SEVA collection of broad host range gram-negative plasmids [44].

The toolkit of modular DNA parts and modified pSEVA plasmid vectors for cloning these parts allowed the team to engineer *K. rhaeticus* to respond to externally applied AHL chemical levels as the cells grew pellicles. This was used to demonstrate spatial patterning, by inducing the production of red fluorescence at one only side of a growing sheet of BC and only in the most recently produced material layers. The red fluorescence module was then swapped for another that expressed a synthetic RNA-based silencing system and this was used to repress UPGase, a chromosomal gene essential for the synthesis of BC. The result was an engineered strain that stopped producing a pellicle when a high concentration of AHL was externally applied [41].

A follow-up paper from the same team later showed that induction of gene expression from plasmids in these cells could be directed by the presence of other engineered cells grown in co-culture [45]. Strains engineered in Florea et al. (2016) to respond to the AHL inducer and produce red fluorescent protein (RFP) were partnered with new strains engineered to constitutively express an enzyme (LuxI) that synthesizes AHL [46]. These new strains (known as ‘sender’ cells) secrete AHL molecules into the media and when they are at high concentration and close proximity to the AHL-controlled cells (‘receiver cells’) they trigger them to produce RFP. The authors showed that these two cells could be co-cultured to grow BC pellicles that trigger their own red fluorescence and more importantly could be used to grow materials that self-sense the boundaries engineered cell populations and trigger gene expression only at these sites [45]. This foundational advance holds future promise for growing BC-based materials with genetically-encoded self-patterning.

The modular DNA toolkit first developed by Florea et al. was expanded by Teh et al. in 2019 [47]. Using the same plasmids and cloning framework, they significantly expanded the available number of characterized modular DNA parts by the addition of multiple new gene part libraries. These included new constitutive promoters, new terminator sequences, parts encoding C-terminal peptide tags that tune the degradation rate of proteins and ribosome-binding site (RBS) sequences that tune the strength of mRNA translation. They also added another externally inducible promoter system, P_BAD_, that responds to high concentrations (4%) of the sugar arabinose and has a much tighter off-state than the previously described AHL-inducible system. Importantly, the authors showed that the cloning toolkit and their new modular DNA parts worked not just in *K. rhaeticus* but also in commonly-used *K. xylinus* and *K. hansenii* strains too [47].

To demonstrate the use of the DNA tools, they repeated the achievement of Yadav et al. in producing the chitin-cellulose copolymer by the introduction of 3 *Candida albicans* genes encoding the metabolic pathway (Figure 1) [22]. The synthetic biology approach of using modular DNA parts allowed them to construct variants of the pathway-expressing plasmid with either low or high expression of the three enzymes. They showed that altering enzyme expression (e.g., by exchanging the promoter used) tuned the amount of incorporation of chitin into the cellulose polymer.

Finally in a further display of the versatility of working with a modular DNA toolkit, they showcased using CRISPR-mediated inhibition (‘CRISPR inhibition’) of genome-encoded genes in *K. hansenii.* A plasmid was constructed expressing both the mutant ‘dead’ version of Cas9—dCas9—and a single guide RNA (sgRNA) that directs this protein to bind to the *acsAB* gene just downstream of its promoter [47]. A small but measurable decrease in cellulose production was observed, consistent with a mechanism whereby the dCas9:sgRNA complex bound to the *acsAB* promoter DNA, blocks transcription and thus inhibits expression of the main cellulose synthase machinery in these cells. By bringing CRISPR-based DNA tools into the genetic toolkit, their work opens up many new opportunities for rapidly engineering and modifying BC-producing bacteria.

Parallel work by researchers in China has also demonstrated the use of CRISPR-inhibition (CRISPRi) to control native gene expression in *K. xylinus* [48]. This stemmed from work analyzing the transcriptome of *K. xylinus* CGMCC 2955, a strain with a very close genome sequence to that of *K. rhaeticus* [49]. Transcriptional analysis identified that the native gene *galU* would be a good target for controlling the metabolic flux into BC production and so may be a way to alter BC production rates and change the porosity and crystallinity of the cellulose produced from this strain. The authors then constructed modular DNA plasmids expressing dCas9 and sgRNAs that direct dCas9 binding to promoter regions and so inhibit the transcription of the targeted genes. Several metabolic genes were targeted and verified to be downregulated by RT-PCR. When they used this approach to modulate expression of the *galU* gene they succeeded in producing BC with increased porosity and reduced crystallinity as *galU* is repressed [48].

Not content with just using CRISPR tools to regulate the native genes on the *K. xylinus* chromosome, the same team have recently developed a modular DNA approach to delete genomic regions in their strain. By transiently expressing the Lambda Red enzymes that are used to enhance homologous recombination of DNA in *E. coli* [50], they were able to direct homology-flanked DNA into *K. xylinus* chromosome in order to delete *gdh*, the gene encoding glucose dehydrogenase*,* which is key to the production of the by-product gluconic acid during BC growth. Importantly, they included a recombinase into their genome-integration workflow so that the antibiotic selection gene used in the process could be removed after insertion, via recombination-mediated deletion between flanking recombination sites [27]. Genomic disruption of *gdh* did indeed prevent gluconic acid production from their strain and when two enzymes that facilitate glucose utilization were then introduced and expressed from plasmids, the final engineered cells showed improved BC yield compared to the un-engineered control [27].

Improved production of BC via a synthetic biology approach was also recently demonstrated by the Korean team whose metabolic engineering efforts described above had pushed *K. xylinus* BC yields to record levels. They developed and characterized a library of RBS modular parts similar to those described by Teh et al. These were then used with modular DNA assembly to create a set of plasmids each expressing an operon of the three key genes for UDP-glucose production; *pgm, galU* and *ndp* (Figure 1) [51]. By varying the strength of the RBS part used to define the translation level of each gene they were able to find an optimal combination of enzyme expression levels to boost BC titer up to 5.28 g/L for static cultures.

## 5. BC-Based Materials with New Functionalities

**Figure 2:**
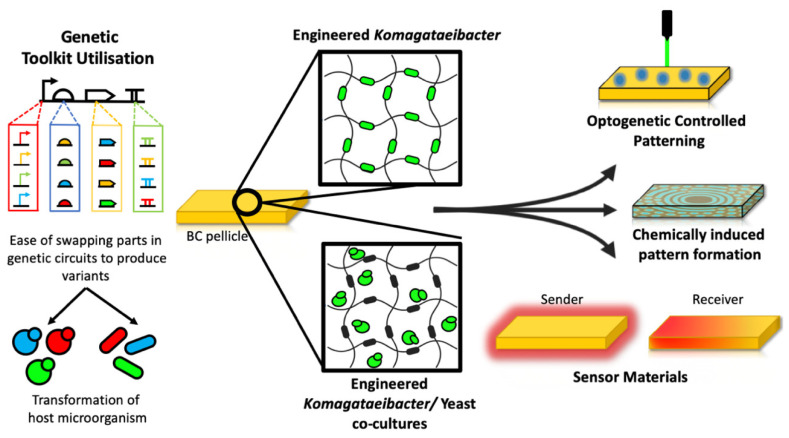
Summary of synthetic biology approaches used to produce functional, living BC-based materials. Left: modular DNA parts (promoters, ribosome-binding site (RBS), coding sequence (CDS) and terminators) from synthetic libraries are assembled together to make gene expression constructs that are transformed into *Komagataeibacter* (rods) or yeast (circles). Centre: engineered cells are cultured to grow bacterial cellulose (BC).pellicles with a network of cellulose fibers containing within them the cells expressing synthetic gene constructs. Right: the living cells within the BC pellicle respond to light, chemicals or diffusible signaling molecules and in response create patterns in the material.

Synthetic biology has already shown that DNA engineering is a route to achieving higher titers of BC from *Komagataeibacter* and to tune its key material properties. But going forward, the synthetic biology approach likely offers greater opportunities for adding entirely new functional material properties to the BC, enabling researchers to be able to grow new ‘smart’ materials that interact with their environment (Figure 2). The work of Yadav et al. inspires us to think about how engineering and modification of the bacteria can produce new materials, such as their chitin-cellulose copolymer, which can be degraded in vivo and thus offers promise for surgical implants [23]. Recently a team demonstrated that glucose monomers chemically conjugated to a fluorescent moiety (6-carboxyfluorescein) could be incorporated by the cellulose synthase machinery of *K. sucrofermentans* to produce a copolymer with inherent fluorescent properties [52]. Although no genetic modification of the bacteria was performed for this (they simply fed the modified sugar to the bacteria) it highlights the diversity of copolymers that may be possible to make via the cellulose synthase machinery. Future use of synthetic biology could engineer the bacteria to make other modified sugar monomers within the cells, for example ones with reactive groups, that lead to copolymers with novel properties.

Along with copolymers, another promising opportunity is composite biosynthesis by the bacteria, where a second polymer is synthesized alongside the cellulose. Already, Fang et al. (2015) have used genetic engineering to demonstrate this possibility. They introduced the gene for curdlan synthesis from Agrobacterium into *K. xylinus* via expression from a plasmid and this led to intracellular UDP-glucose being polymerized to make cellulose fibers and curdlan in parallel from the engineered cells [53]. This did not dramatically alter the material properties but highlights the possibility of using synthetic biology to achieve further co-synthesized BC-based composites. Many other bacteria, including *E. coli,* produce strong biofilms that are composites of small amounts of cellulose and other biopolymers like lipopolysaccharides (LPS) and amyloid protein fibers [54,55]. If the genes encoding the molecular machinery to make and secrete these polymers can be engineered into *Komagataeibacter*, then a variety of interesting biological composites of BC could be possible. More importantly, the relative composition of the different polymers to these materials (e.g., protein fibers vs cellulose fibers) could be tuned at the DNA level by using different strength promoter and RBS parts in the genetic programs or by having external control (e.g., via chemical inducers like AHL) of the gene expression of the enzymes performing the polymer synthesis.

Synthetic biology teams working in *E. coli* have in recent years reported the biosynthesis of several interesting new materials with functionalities by modifying the DNA that encodes curli; a fibrous amyloid protein polymer secreted by some strains [56,57]. As the polymer itself is modular, consisting of hundreds of copies of the curli protein that self-assemble into a fiber, it is well-suited to synthetic biology as the DNA encoding the protein can be rationally engineered to fuse new protein modules that add on functions, for example metal binding domains or underwater adhesive domains [58,59].

Unfortunately, protein secretion is not well understood in BC-producing strains, making it hard to use synthetic biology to engineer *Komagataeibacter* to secrete proteins alongside the cellulose fibers. Future work should focus on solving this challenge, as it will not only enable composites of cellulose and protein polymers to be made but will also open up many possibilities for secreting enzymes and other proteins which can attach among the BC fibers in a growing material and modify the fibers or do other tasks [60].

The many possibilities of using synthetic biology to program protein secretion into growing bacterial cellulose were highlighted recently in work that took an alternative ‘co-culture’ approach to making BC-based materials with new functional properties. Taking inspiration from the symbiotic culture of bacteria and yeast (SCOBY) that is used traditionally to ferment kombucha tea [61], the authors devised a symbiotic culture of *K. rhaeticus* and *S. cerevisiae* yeast that grows efficiently on sucrose and produces thick BC pellicles that contain within them both the bacteria and the yeast cells [62]. The synthetic biology was in this case not applied to BC-producing bacteria but instead to the co-cultured yeast. As *S. cerevisiae* is a well-established advanced host for synthetic biology this opens up many new engineering possibilities not yet achievable from just modifying *Komagataeibacter*. For example, *S. cerevisiae* is capable of programmable protein secretion via fusion of genes to the leader sequence of its native alpha mating factor.

The yeast cells were genetically modified to display cell-surface cellulose binding proteins to have them bind among the BC fibers as the material was produced. DNA programs were then added to make the yeast secrete a variety of enzymes that attach themselves to the cellulose and perform reactions. This was used to weaken the material (by secretion of cellulases) or give the material catalytic properties, for example, by having it populated with pollutant-degrading enzymes. Live yeast cells trapped among the BC could also be engineered to sense environmental cues from within the material, such as chemicals that are found in wastewater, peptide hormones and even sense light. In response to this sensing the cells were engineered to secrete enzymes or to produce fluorescent and luminescent proteins that can be visibly detected from the grown material. The overall result is a way to grow bacterial cellulose and use synthetic biology to program a variety of multifunctional properties to be present into the produced material; from BC materials that break down pollutants, to BC-based ‘photographs’ that glow in response to light stencils (Figure 2) [62].

Hopefully future work in achieving engineerable protein secretion from *Komagataeibacter* species will allow similar or even more impressive achievements through the use of synthetic biology in the bacteria. Cellulose materials that are grown with enzymes or other proteins attached throughout are likely to offer many opportunities in diverse application areas; from water filters designed with proteins embedded throughout the material to bind and degrade target contaminants, through to scaffold materials for regenerative medicine that have growth factors patterned in specific locations to promote proliferation and differentiation of stem cells.

## 6. Concluding Remarks

In conclusion, the last five years have seen a burst of new approaches to modify BC-producing bacteria via genetic interventions. Metabolic engineering guided by genome-scale metabolic models has been applied to achieve record titers of BC from *K. xylinus* strains and even modify the genomic DNA of these strains to reduce their chance of deleterious mutations. Modular DNA toolkits that enable more radical, rapid and yet simplified strain engineering via synthetic biology have been developed for *K. rhaetcius* and then expanded by others with more DNA parts and shown to also work in *K. xylinus* and *K. hansenii* strains. CRISPR-based regulation and genome modification tools are also now part of the toolkit for engineering these bacteria in order to modify the materials produced.

As more people in academic research, industry, healthcare biotechnology, as well as those in the arts and textiles become more familiar with bacterial cellulose as an exciting and useful material, there will be an increasingly large community of people looking to diversify what BC can be made to do using accessible tools. The rise of 3D printing as a readily available method that no longer requires specialist skills and expensive equipment for people to use should be inspiration for those developing synthetic biology toolkits where the aim of the field is to make engineering cells faster, cheaper and more reliable. Allowing a wider community to develop and test DNA parts and programs for modifying BC production and adding new functions to BC promises to create a richer field that can ensure that materials based on bacterial cellulose become ever more diverse and have a higher chance of becoming useful products that have impact on many different sectors.
